# Evaluation of the rural response system intervention to prevent violence against women: findings from a community-randomised controlled trial in the Central Region of Ghana

**DOI:** 10.1080/16549716.2019.1711336

**Published:** 2020-01-14

**Authors:** Deda Ogum Alangea, Adolphina A. Addo-Lartey, Esnat D. Chirwa, Yandisa Sikweyiya, Dorcas Coker-Appiah, Rachel Jewkes, Richard M. K. Adanu

**Affiliations:** a Department of Population, Family and Reproductive Health, School of Public Health, University of Ghana, Accra, Ghana; b Department of Epidemiology and Disease Control, School of Public Health, University of Ghana, Accra, Ghana; c Gender and Health Research Unit, South African Medical Research Council, Pretoria, South Africa; d Gender Studies and Human Rights Documentation Centre, Accra, Ghana

**Keywords:** Intimate partner violence, violence against women, randomised control trial, community intervention, the rural response system, Ghana

## Abstract

**Background**: Intimate partner violence (IPV) affects one in three women globally and undermines women’s human rights, social and economic development, and health, hence the need for integrated interventions involving communities in its prevention.

**Objective**: This community-randomised controlled trial evaluated the Rural Response System (RRS) intervention, which uses Community Based Action Teams to prevent IPV by raising awareness and supporting survivors, compared to no intervention.

**Methods**: Two districts of the Central Region of Ghana were randomly allocated to each arm. Data were collected by repeated, randomly sampled, household surveys, conducted at baseline (2000 women, 2126 men) and 24 months later (2198 women, 2328 men). The analysis used a difference in difference (DID) approach, adjusted for age and exposure to violence in childhood.

**Results**: In intervention communities, women’s past year experience of sexual IPV reduced from 17.1% to 7.7% versus 9.3% to 8.0% in the control communities (DID = −9.3(95%CI; −17.5,−1.0), p = 0.030). The prevalence of past-year physical IPV among women in the intervention communities reduced from 16.5% to 8.3% versus 14.6% to 10.9% in the controls (DID = −4.2(−12,3.6), p = 0.289). The prevalence of severe IPV experienced by women reduced from 21.2% to 11.6% in intervention versus 17.3% to 11.4% in controls (DID = −3.7(−12.5,5.1), p = 0.408). The direction of impact of the intervention on violence perpetrated by men was more towards a reduction but changes were not statistically significant. Emotional IPV perpetration was significantly lower (DID = −15.0(−28.5, −1.7), p = 0.031). Women’s depression scores and reports of male partner controlling behaviour significantly also reduced in the intervention arm compared to those in the control arm (DID = −4.8(−8.0,−1.5), p = 0.005; DID = −2.7(−3.3,−1.0), p = 0.002, respectively).

**Conclusion**: Our findings indicate that the RRS intervention reduced women’s experiences of IPV, depression, and partner controlling behaviour and some evidence of men’s reported reductions in the perpetration of IPV. The RRS intervention warrants careful scale-up in Ghana and further research.

## Background

Violence against women (VAW) is a threat to the rights and wellbeing of women globally. The greater proportion of VAW is perpetrated by intimate partners. Intimate partner violence (IPV) includes emotional, economic, physical, verbal, and sexual abuse of women by a current or ex-husband or boyfriend. It is a widely recognised phenomenon that affects over 30% of women with widespread regional variations across the world. Sub-Saharan Africa currently has the highest IPV burden [1].

The consequences of VAW transcend the negative physical, mental, psychosocial and economic impact on the victims to impact their children and society at large. Evidence from 42 Demographic and Health Surveys showed that children born to women who have been exposed to IPV experience more violence themselves and have poorer nutritional outcomes [2]. Additionally, women exposed to IPV have less access to contraception and reproductive health services, skilled birth attendance and general healthcare services. Such poorer access to health care, together with a higher prevalence of morbidity and mortality, results in a very substantial personal, household and wider economic burden on societies [3].

Even with the high prevalence and the negative impact of VAW, there are relatively few interventions proven to reduce VAW. A review of the evidence on interventions to prevent violence against women and girls (VAWG) carried out by Ellsberg et al. showed that interventions that were more effective in reducing VAW often included participatory group sessions, promoting better communication, were multi-component, often engaged multiple stakeholders, and promoted shared decision-making by couples. Some worked through community mobilisation, usually engaging men and women in communities as change agents, and greater economic empowerment of women, with and without gender-transformative elements of the intervention, often reduced VAW [4]. There were two evidence-based trials of interventions that sought to work across the community through approaches that sought to change social norms on gender and violence. These evaluated the SHARE (Safe Homes and Respect for Everyone) intervention and the SASA! Activist Kit for Preventing Violence against Women and HIV. Both were conducted in Uganda and assessed the impact of IPV at the community level [5,6]. In SHARE, there was a statistically significant decrease in IPV experienced by women at the community level, whiles SASA! reported a reduction in sexual and physical violence experienced by women, although reduction did not reach statistical significance. These trials showed the promise of this modality of intervention but neither achieved statistically significant changes reported by men.

In Ghana, the Gender Studies and Human Rights Documentation Centre (GSHRDC) designed the Rural Response System (RRS) [7] intervention in 2002 to reduce violence against women and girls following findings of a survey on VAWG conducted in 1988 [8]. Since then (2002–2008), the RSS intervention was implemented in four regions (and 18 communities) of Ghana. Although programme reports showed positive improvements in the reduction of VAWG in pilot districts, no formal impact assessment of this intervention had been conducted. As part of a global search for interventions that work to reduce VAWG, the RRS was tested in Ghana for the first time with funding from UKAid from the UK Government via the ‘What works to prevent violence against women and girls?’ Global programme. The trial was a collaboration between the University of Ghana, Gender Studies and Human Rights Documentation Centre and South African Medical Research Council. This paper evaluates the impact of the RRS intervention on intimate partner violence in four districts of the Central Region of Ghana.

## Methods

### Intervention description – the rural response system (RRS)

Given the complex interplay of societal and institutional factors in intimate partner violence which operate at the individual, interpersonal, community, and societal levels, the RRS works with a broad range of stakeholders within the community for effective change to be achieved. The overall aim of the intervention was to reduce the incidence of all forms of violence experienced by women and violence perpetrated by men and protect women’s human rights through state and community-based structures. The objectives of the RRS are to increase knowledge on VAW; change individual and community attitudes towards gender equality and violence; positively change social and gender norms and behaviours that perpetuate gender inequality and VAW; provide counseling and support to couples affected by IPV and other victims of VAW, and assist victims to seek redress from state institutions; to develop a referral system between the community-based response systems and state agencies to encourage a consistent and coordinated response; and to strengthen appropriate traditional systems of resolution of VAW.

These objectives of the RRS are achieved through the institution of Community Based Action Teams (COMBATs) within the communities, trained and facilitated by the Gender Centre, and supported in their work over 18 months. The COMBAT members are respected male and female members of the community, nominated by the community to play key roles in realising intervention goals. Six people were chosen per community and the study was implemented in 20 communities. The teams usually worked together when undertaking community sensitisation and awareness-raising, but members could also work individually. They were encouraged to use every opportunity, such as community festivals and meetings, weddings, funerals, Parents Teachers Association meetings, membership meetings of social associations, religious groupings, and other meetings of family and friends to carry out this role. They would use these opportunities, for example, to share information on wives’ property rights after bereavement and the importance of wills, to talk about how to share work in the home and have a non-violent marriage, or to argue against child neglect. They would also provide counselling for couples referred to them because they were known to be experiencing violence. More detail about criteria for COMBAT selection, training components and implementation strategy of the RRS has been previously described [9]. The RRS intervention was implemented over an 18-month period.

The RRS also provided training for staff of some State Agencies, from the police, health, social welfare, Commission on Human Rights and Administrative Justice and National Commission on Civic Education. They also provided training for some other community-based organisations and had regular meetings with community traditional and religious leaders and other stakeholders around their roles, responsibilities, and messages in relation to VAW.

### Study design and setting

This trial was an unblinded community-randomised controlled study carried out in four districts of the Central Region of Ghana: two coastal districts (Abura and Komenda), and two inland districts (Agona and Upper Denkyira). Our trial protocol has been registered and is available on ClinicalTrials.gov (NCT03237585). Most (63%) of the region is rural and a population density of about 215 inhabitants per square kilometre [10,11]. About 50% of adults in the region are literate with higher male literacy than females (69.8% vs 46.3%). The Central Region is predominantly Akan speaking (82.0%) with Fante as the indigenous dialect of most districts in the region. Agriculture (cocoa, oil palm, pineapple, grains) and fishing are the primary livelihoods and employ more than two-thirds of the workforce in many districts [10].

### Study population and sampling

Districts in the Central Region within which previous VAW research had been carried out were excluded. Two inland and two coastal districts were purposefully selected from the eligible districts as study sites in order to allow for at least one-district-wide geographical buffer separating the designated sites. The districts were randomly assigned intervention or control through a blind draw of the four selected districts (district names were written on paper and placed in a bag) by the study statistician in Pretoria. Ten localities were randomly selected in each district resulting in a total of 40 localities considered as clusters in this study. Each cluster (localities) contained one or more enumeration areas (EAs) of varying sizes. The second layer of stratification by gender was done using EAs, (resulting in 42 male-designated and 38 female-designated EAs) for the quantitative surveys. The stratification of the EAs was to ensure the physical separation of male and female designated EAs during data collection to ensure the safety of women and researchers in accordance with WHO safety recommendations for studies on VAW [12]. A proportionate sampling of households within EAs was carried out based on EA size, followed by a computerised random selection of households from a list of households in each EA. The list of EAs was obtained from the Ghana Statistical Service but listing of households was conducted by the study. Only one eligible male or female was interviewed per household. Balloting was used to select a participant in situations where more than one eligible adult was available in the household.

**Figure 1.**
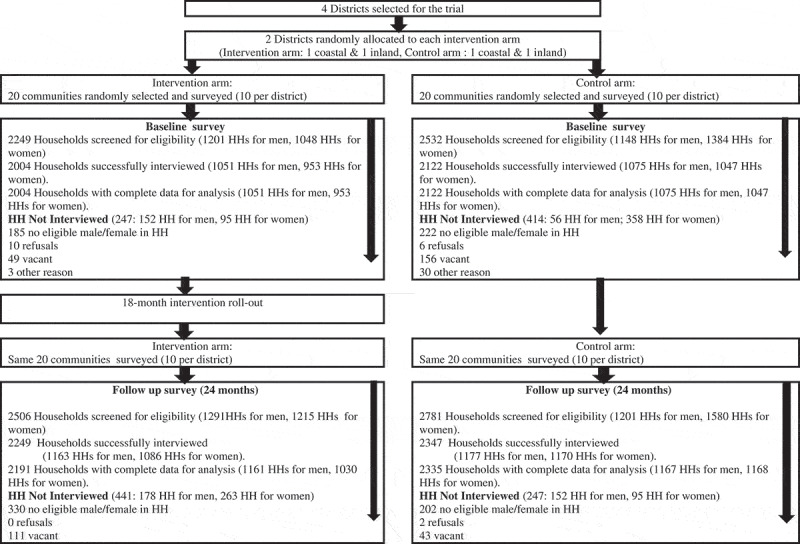
The RRS trial consort diagram

Sample size for this trial was determined using the method of Hayes and Bennet [13] and based on IPV estimates (34.9% IPV experience) from the 2008 Ghana DHS [11], an expected reduction of IPV by 30% in intervention arms, 90% power and significance level of 0.05 (two-sided). Factoring in 15% oversampling to allow for incomplete questionnaires, the estimated sample size was 1640 per trial arm (820 currently partnered males and 820 currently partnered females per trial arm at baseline and at post-intervention). We further oversampled by approximately 20% to ensure that the minimum sample of 3280 participants that had to be partnered in the past year was obtained for our primary outcome analysis. A total of 4,148 women (pre-intervention = 2000; post-intervention = 2198) and 4,454 men (pre-intervention = 2,126; post-intervention = 2,328) were interviewed. At pre-intervention, 1877 out of 2000 women and 1973 out of 2126 men sampled were currently partnered. At post-intervention, 1979 out of 2198 and 2200 out of 2328 men were currently partnered. More details on participation are shown in the trial consort diagram in Figure 1.

Eligibility was based on residence within the community for a least a year and for men having an age of 18 years or older, or for women, being 18-to-49 years old. Selected participants had to be able to communicate in the main languages of the study (English, Twi, and Fante) and not suffer from a severe mental deficit (learning difficulty, severe mental illness or intoxication) that affected their ability to consent. At endline, participation in the study was solely based on the eligibility of individuals within randomly selected households taking no cognisance of prior involvement in the pre-intervention survey. We did not ask if participants had participated in the pre-intervention survey since the intervention was both implemented and evaluated at the community level. Thus, all adults resident in the community had equal chances of coming into contact with intervention as well as being selected for surveys upon meeting the eligibility criteria.

### Data collection tools and procedures

Although qualitative and quantitative methods were used to understand the context of VAW, estimate the burden and evaluate the impact of the RRS on men and women in participating districts, only the quantitative components of the trial are presented here.

The survey tool consisted of questions that had been validated in a similar trial in South Africa [14,15] and included standard measurements developed by the World Health Organisation for its multi-country study on domestic violence against women [16]. Questionnaires were initially translated into local dialects (Fante and Twi) by an independent consultant and then edited by bi-lingual members of the project team at the University of Ghana. The revised translations were then independently re-translated by another consultant who had not seen the English version of the questionnaire. The project team then used a consensus-building translation method to finalise the translated questionnaire which was pretested in a population similar to that of the study population.

All data were collected using interviewer-administered face-to-face interviews recorded onto personal digital assistants. The trial followed ethical principles outlined by the World Medical Association Declaration of Helsinki [17], and the Belmont report [18]. Ethical approval for this trial was obtained from the Institutional Review Board at the Noguchi Memorial Institute for Medical Research at the University of Ghana (#006/15-16) and the South African Medical Research Council’s Ethics Committee (EC031-9/2015). Eligible participants were provided with informed consent, assured of confidentiality, anonymity, and minimised risk of participation. Consenting participants (thumb printed or signed) were interviewed in their homes in their preferred local dialect. Further detail on the methods used in this trial is presented elsewhere [9]. The Pre-intervention survey was conducted from January till May 2016 and the post-intervention survey took place from January till May 2018.

### Study outcomes

The primary and secondary outcomes of the trial are summarised in Appendix 1. These were assessed 24 months post-baseline and pre-specified in the trial protocol [9] The primary outcomes of this trial were: (1) prevalence in past year physical IPV experience (women) and perpetration (men); (2) prevalence in past year sexual IPV experience (women) and perpetration (men); (3) prevalence of severe physical/sexual IPV experience (women) and perpetration (men). Any participant who responded affirmatively to any one of the five questions on physical IPV or three questions on sexual IPV in the past 12 months was considered to have experienced IPV (if female) or perpetration IPV (if male). Severe IPV was assessed by combining the five physical and three sexual IPV questions. Women were deemed to have experienced (and men perpetrated) severe IPV if a participant responded positively to two or more items, or else responded: few or many, to any single item from these eight questions.

The secondary outcomes included: (1) change in gender attitudes; (2) change in individual gender attitudes (3) change in perceived social norms; (4) change in controlling behaviour of male partners; and (5) change in depression.

### Statistical analysis

Statistical analysis used Stata version 15. The approach to analysis was an intention to treat, thus, respondents were included in the arm of analysis according to where they lived, whether or not they reported contact with the intervention. Among the partnered women and men, primary outcomes were derived from items on physical or sexual IPV experience (women)/perpetration (men). The participant was classified as having experienced/perpetrated physical IPV if they responded positively (once, few times or many times) to any of the 5 items on physical IPV. Similarly, a participant was classified as having experienced/perpetrated sexual IPV if they responded positively to any of the 3 items on sexual IPV. Severe IPV was assessed by combining the five physical and three sexual IPV questions. Participants were deemed to have experienced (women) or perpetrated (men) severe IPV if a participant responded positively to two or more items, or else responded: few or many, to any single item from these eight questions.

For secondary outcomes measured using scales such as gender attitudes, social norms, and controlling behaviour, we derived additive scores from the items in the scales after testing for internal consistency using Cronbach alpha. Before deriving scores, we examined the data for missing item responses. No imputation of missing data was done as there were no missing data in any of the items for the different scales.

Before testing the impact of the intervention on the outcomes at endline, we assessed for any differences in the primary outcomes and socio-demographic factors at baseline. Descriptive statistics were determined for all measured outcomes and summarised in tables comparing study arms. Since the intervention was done at community (cluster) level, we derived summaries for all outcomes at the cluster level (percentages for binary outcomes and means for continuous outcomes). To account for differences in primary outcomes at baseline between arms, we used the difference in difference (DID) method to assess the impact of the intervention at endline. The DID looks at the differences in outcomes at endline between intervention and control arms, taking into account the difference between arms at baseline and the change within arms from baseline to endline. For all outcomes where the hypothesised direction of change due to the intervention was a decrease in mean/percentage (see Appendix 1), a negative value of the model coefficient (adjusted DID) implied a better outcome in the intervention arm relative to the control arm. Similarly, for all outcomes where the hypothesised direction of change due to the intervention was an increase in mean/percentage, a positive value of the model coefficient (adjusted DID) implies a better outcome in the intervention arm relative to the control arm. The selection of adjustment variables was based on pre-determined risk factors such as childhood violence exposure/experience, education level and food insecurity [19,20], but we also aimed at achieving parsimonious models. All models were adjusted for mean age at the cluster level and final models for IPV were adjusted for age and exposure to violence in childhood (witnessing the abuse of the mother in childhood).

## Results

The response rate for this study was 99.7% at the pre-intervention interview round (16 eligible participants declined participation) and 99.9% at post-intervention (2 persons declined participation).

### Intervention coverage

**Figure 2.**
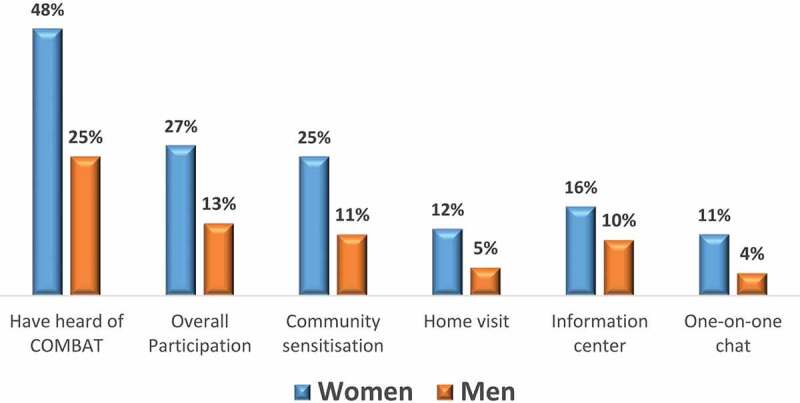
Coverage of the RRS trial

After 18 months of implementation of the intervention, nearly one-half (48%) of women and a quarter (25%) of men in the two intervention districts had heard of the intervention (the term ‘COMBAT’). One-fourth of women and one in ten (11%) men had participated in a sensitisation activity, while 16% of women and 10% of men had received education on VAW from the information centre messages (a megaphone broadcast by COMBATs at dawn). In addition, one in eight (12%) women and one in twenty men (5%) reported having received a home visit. Figure 2 shows the participation of men and women in different intervention activities.

### Socio-demographic characteristics

The samples of male and female participants were very similar in their age distributions and the level of education across the two data collection points and study arms. At Pre-intervention, 85.9% and 87.4% of women in the control and intervention arms were married or in a relationship, and 85.4% and 86.3% of men in the control and intervention arms (Table 1 & Table 2). In the post-intervention survey, 86.0% of women in the control arm and 84.9% in the intervention, and 92.5% of men in the control arm and 84.5% in the intervention were married or in relationships, these differences were significant for the men at this time point (p = 0.04).

**Table 1.** Socio-demographic characteristics of female participants in the RRS trial pre- and post-intervention

	Pre-intervention			Post-Intervention		
	Control	Intervention		Control	Intervention	
	(N = 1048)	(N = 952)		(N = 1168)	(N = 1030)	
Characteristic	n (%)	n (%)	p-value	n (%)	n (%)	p-value
**Age of respondents**						
<20 yrs	55 (5.2)	53 (5.6)	0.682	80 (6.8)	56 (5.4)	0.526
20–24 yrs	198 (18.9)	178 (18.7)		248 (21.2)	203 (19.7)	
25–29 yrs	202 (19.3)	216 (22.7)		231 (19.8)	203 (19.7)	
30–34 yrs	198 (18.9)	161 (16.9)		174 (14.9)	184 (17.9)	
35–39 yrs	148 (14.1)	128 (13.4)		160 (13.7)	143 (13.9)	
40–44 yrs	124 (11.8)	113 (11.9)		125 (10.7)	118 (11.5)	
45–49 yrs	123 (11.7)	103 (10.8)		150 (12.8)	123 (11.9)	
**Marital status**						
Married	579 (55.2)	489 (51.4)	0.25	525 (44.9)	499 (48.4)	0.365
Separated/Divorced/No relationship	148 (14.1)	120 (12.6)		164 (14.0)	156 (15.1)	
Not married but in relationship	321 (30.6)	343 (36)		479 (41.0)	375 (36.4)	
**Educational level**						
None	218 (20.8)	216 (22.7)	0.651	226 (19.3)	211 (20.5)	0.934
Primary	234 (22.3)	225 (23.6)		270 (23.1)	246 (23.9)	
Junior High School	468 (44.7)	429 (45.1)		516 (44.2)	446 (43.3)	
Senior High School or Higher	128 (12.2)	82 (8.6)		156 (13.4)	127 (12.3)	
**Worked or earned income in past 3 months**	596 (57)	657 (69.1)	0.041	677 (58.0)	583 (56.6)	0.531
**Years lived in the community**						
<5 yrs	241 (23.0)	154 (16.2)	0.018	245 (21.0)	155 (15.0)	0.018
5–9 yrs	132 (12.6)	109 (11.4)		187 (16.0)	112 (10.9)	
10–19 yrs	198 (18.9)	239 (25.1)		238 (20.4)	245 (23.8)	
20–29 yrs	260 (24.8)	295 (31.0)		260 (22.3)	308 (29.9)	
≥30 yrs	217 (20.7)	155 (16.3)		238 (20.4)	210 (20.4)	
**Food insecurity**						
Low	314 (30.0)	237 (24.9)	0.112	574 (49.4)	452 (43.9)	0.006
Moderate	330 (31.5)	359 (37.7)		286 (24.6)	375 (36.4)	
Severe	404 (38.5)	356 (37.4)		303 (26.1)	203 (19.7)

**Table 2.** Socio-demographic characteristics of male participants in the RRS trial pre- and post-intervention

	Pre-intervention			Post-intervention		
	Control	Intervention		Control	Intervention	
	(N = 1075)	(N = 1051)		(N = 1167)	(N = 1161)	
Characteristic	n (%)	n (%)	p-value	n (%)	n (%)	p-value
**Age of respondents**						
<20 yrs	40 (3.7)	40 (3.8)	0.69	49 (4.2)	85 (7.3)	0.227
20–24 yrs	141 (13.1)	142 (13.5)		164 (14.1)	179 (15.4)	
25–29 yrs	170 (15.8)	154 (14.7)		191 (16.4)	172 (14.8)	
30–34 yrs	135 (12.6)	125 (11.9)		161 (13.8)	154 (13.3)	
35–39 yrs	141 (13.1)	115 (10.9)		165 (14.1)	134 (11.5)	
40–44 yrs	110 (10.2)	114 (10.8)		108 (9.3)	111 (9.6)	
45–49 yrs	81 (7.5)	81 (7.7)		82 (7.0)	77 (6.6)	
≥50 yrs	257 (23.9)	280 (26.6)		247 (21.2)	249 (21.4)	
**Marital status**						
Married	597 (55.5)	674 (64.1)	0.219	636 (54.5)	628 (54.1)	0.040
Separated/Divorced/No relationship	157 (14.6)	165 (15.7)		87 (7.5)	178 (15.3)	
Not married but in relationship	321 (29.9)	212 (20.2)		444 (38.0)	355 (30.6)	
**Educational level**						
None	282 (26.2)	126 (12.0)	0.118	270 (23.1)	77 (6.6)	0.059
Primary	171 (15.9)	188 (17.9)		172 (14.7)	219 (18.9)	
Junior High School	283 (26.3)	406 (38.6)		365 (31.3)	445 (38.3)	
Senior High School or Higher	339 (31.5)	331 (31.5)		360 (30.8)	420 (36.2)	
**Worked or earned income in past 3 months**	605 (66.1)	690 (76.5)	0.019	798 (79.7)	917 (89.5)	0.006
**Years lived in the community**						
<5 yrs	76 (7.1)	170 (16.2)	<0.003	70 (6.0)	152 (13.1)	<0.001
5–9 yrs	72 (6.7)	119 (11.3)		74 (6.3)	124 (10.7)	
10–19 yrs	142 (13.2)	226 (21.5)		173 (14.8)	269 (23.2)	
20–29 yrs	278 (25.9)	250 (23.8)		356 (30.5)	316 (27.2)	
≥30 yrs	507 (47.2)	286 (27.2)		494 (42.3)	300 (25.8)	
**Food insecurity**						
low	365 (34.0)	422 (40.2)	0.077	384 (32.9)	582 (50.1)	0.020
moderate	197 (18.3)	284 (27.0)		306 (26.2)	324 (27.9)	
severe	513 (47.7)	345 (32.8)		477 (40.9)	255 (22.0)	

More women and men in the intervention district had worked or earned income in the past three months compared to their counterparts in control districts at pre-intervention (69.1% vs. 57%, p < 0.05 for women; 66.1% vs. 77.5% p = 0.019 for men). These types of differences were also seen for men post-intervention (79.7% vs. 89.5% p = 0.006 for men). Women and men in control districts, compared to intervention districts, recorded more severe household food insecurity post-intervention (26.1% vs. 19.7% p = 0.006 for women, 40.9% vs. 22.0%, p = 0.02 for men), this was not found pre-intervention for women and for men there was a non-significant trend in this direction pre-intervention (p = 0.07). Other details of the socio-demographics of women are shown in Table 1 and of men in Table 2.

### Primary outcomes

Table 3 presents the intervention effects on the primary outcomes of the study. Pre-intervention estimates of past year sexual or physical IPV experience were higher among women in intervention communities compared to controls (16.5% vs. 14.6% for physical; 17.1% vs. 9.3% for sexual). Prevalence of physical IPV experience among women reduced from 16.5% to 8.3% post-intervention while a reduction from 14.6% to 10.9% was observed among controls. Sexual IPV experience among women also reduced from 17.1% to 7.7% and 9.3% to 8.0% among women in intervention and control districts respectively between intervention and controls (DID = −9.3, 95%CI:-17.5–1.0, p = 0.03). Experience of severe forms of physical or sexual IPV by women in intervention districts reduced from 21% to 12% while control districts recorded a reduction from 17% to 11% (DID = −3.7, 95%CI:-12.5–5.1, p = 0.41).

**Table 3.** Differences in primary outcomes evaluation for men and women in the RRS trial

		Baseline	Baseline	End-line	End-line		
Outcome	Study Arm	IPV numbers	mean percentage	IPV numbers	mean percentage	adjusted DID (95% CI)	p-value
**Women’s experience**							
Physical IPV	Control	151/989	14.6	114/1043	10.9		
	Intervention	139/888	16.5	89/936	8.3	−4.2 (−12.0–3.6)	0.289
Sexual IPV	Control	84/989	9.3	71/1043	8.0		
	Intervention	138/888	17.1	85/936	7.7	−9.3 (−17.5- −1.0)	0.030
Severity of IPV (Physical or Sexual)	Control	174/989	17.3	115/1043	11.4		
	Intervention	176/888	21.1	124/936	11.6	−3.7 (−12.5–5.1)	0.408
**Men’s perpetration**							
Physical IPV	Control	108/1008	12.6	141/1122	13.2		
	Intervention	127/965	13.8	131/1078	12.3	−3.6 (−10.6–3.4)	0.318
Sexual IPV	Control	161/1008	15.9	222/1122	20.5		
	Intervention	167/965	17.6	210/1078	20.6	−5.1 (−0.16.3–6.1)	0.368
Severity of IPV (Physical or Sexual)	Control	171/1008	17.6	218/1122	20.0		
	Intervention	201/965	21.7	240/1078	23.3	−4.1 (−15.3–7.1)	0.463

The prevalence of physical or sexual IPV perpetration by men was higher in intervention districts compared to control districts pre-intervention (14% vs. 13% for physical; 18% vs. 16% for sexual). Physical IPV perpetration reduced slightly among men in intervention districts (14% to 12%) but maintained in the control districts (DID = −3.6, 95%CI:-10.6–3.4, p = 0.32), Table 3. Sexual IPV perpetration increased among men in both intervention and control districts post-intervention, although this increase was much less in the intervention compared to control districts. Although the intervention effect showed overall a reduction in IPV perpetration, no significant impact was observed overall (all p > 0.05).

### Secondary outcomes

Women in the intervention arm reported higher levels of depressive symptoms than their counterparts in control districts pre-intervention (mean depression score 19.6 vs 17.4). Post-intervention, a significant reduction was observed in depression levels for women in the intervention districts compared to controls (DID = −4.75; 95% CI:-7.98–−1.52, p = <0.01). Similarly, men in control districts reported higher levels of depression pre-intervention compared to men in control districts. No reduction in depression level among men in intervention districts versus control districts was found (DID = −0.58, 95% CI: −3.58–2.42, p = 0.70), Table 4.

**Table 4.** Differences in depression scores among respondents in the RRS trial

		Baseline	End-line		
Outcome	Intervention Arm	mean score	mean score	Adjusted DID	p-value
**Women**					
Depression score	Control	17.36	16.85		
(high = more depressed)	Intervention	19.62	15.02	−4.75 (−7.98–−1.52)	0.005
**Men**					
Depression score	Control	18.91	17.60		
(high = more depressed)	Intervention	15.11	13.34	−0.58 (−3.58–2.42)	0.703

Women in the intervention arm experienced slightly higher levels of emotional IPV compared to women in the control arm (30% vs 27%) pre-intervention. Post-intervention, women’s experience of emotional IPV reduced (from 30% to 22%) in the intervention arm but was little changed in the control arm (27.3% vs. 26.6%). There was some evidence that the intervention may have had an impact on women’s experience of emotional IPV (DID = −9.6, 95% CI:-20.4- −1.2, p = 0.0.08) although this difference did not achieve conventional statistical significance. Contrarily, the absolute prevalence of men’s perpetration of emotional IPV was higher in both intervention and control arms post-intervention than pre-intervention. However, adjusted estimates show a significantly lower prevalence at endline in the intervention arm relative to the control arm (DID = −0.15, 95% CI:-28.5–−1.7, p = 0.03) at post-intervention.

Women’s report of the controlling behaviour of male partners pre-intervention was slightly higher in the intervention arm compared to the control arm, Table 5. At post-intervention, women in the intervention communities recorded significant reductions in partner controlling behaviour compared to those in the control arm (DID = −2.66, 95% CI: −3.30–−1.02, p = 0.002). No significant changes were observed in men’s reports of controlling behaviour post-intervention, Table 5.

**Table 5.** Differences in emotional/economic IPV and partner controlling behaviour among respondents in the RRS trial

		Pre-intervention	Post-Intervention		
Outcome	Study arm	mean percentage /mean score	mean percentage/mean score	adjusted DID	p-value
**Women**					
Experience of emotional/economic IPV	Control	27.3	26.6		
	Intervention	30.2	21.5	−9.6 (−20.4–−1.2)	0.080
Partner controlling behaviour score (high = more controlling)	Control	19.66	19.56		
	Intervention	21.53	19.01	−2.66 (−3.30–−1.02)	0.002
**Men**					
Perpetration of emotional/economic IPV	Control	21.7	34.1		
	Intervention	31.9	34.3	−15.0 (−28.5- −1.7)	0.031
Controlling behaviour score (high = more controlling)	Control	22.46	22.39		
	Intervention	21.30	21.83	0.50 (−1.22–2.23)	0.562

There was some evidence that women in the control arm may have had somewhat more gender-equitable scores compared to women in the intervention arm pre-intervention (DID = 1.55, 95% CI:-0.26–3.36, p = 0.094), Table 6. Social norms and individual attitudes were similar among women in both arms pre-intervention. Although there were slight improvements in gender attitudes, social norms, and individual attitudes post-intervention, these improvements were not statistically significant. Similar to the women, there were no significant effects of the intervention on men’s gender attitudes and perceived social norms towards gender relations.

**Table 6.** Differences in gender attitudes and norms among respondents in the RRS trial

		Pre-intervention	Post-Intervention		
Outcome	Intervention arm	mean score	mean score	adjusted DID	p-value
**Women**					
Gender attitudes score	Control	15.45	15.68		
(high = equitable)	Intervention	13.92	15.70	1.55 (−0.26–3.36)	0.094
Individual gender attitudes	Control	19.54	19.79		
score (high = equitable)	Intervention	19.05	20.05	0.75 (−0.80–2.30)	0.337
Social norms score	Control	20.04	21.16		
(high = equitable)	Intervention	18.47	20.17	0.58 (−1.11–2.26)	0.498
**Men**					
Gender attitudes score	Control	16.72	15.73		
(high = equitable)	Intervention	16.45	16.66	1.20 (−0.55–2.95)	0.177
Individual gender	Control	25.20	23.85		
attitudes score (high = equitable)	Intervention	24.82	24.68	1.22 (−0.45–2.88)	0.152
Social norms score	Control	19.96	18.49		
(high = equitable)	Intervention	19.90	18.29	−0.14 (−3.342–3.14)	0.932

## Discussion

This trial sought to investigate the impact of the Rural Response System (RRS) intervention in reducing VAW and its effectiveness in enabling women to reduce their exposure to IPV and men to reduce their IPV perpetration. The direction of change in all the primary outcomes in women and men pointed towards a reduction, and this was statistically significant for sexual violence reported by women. In women, there were also statistically significant reductions in partner controlling behaviour and less depression. In men, there was evidence of lower reported perpetration of emotional/economic IPV. Although the overall direction of effect was that of lower IPV, in both intervention and control communities the sexual IPV and economic and emotional IPV reports of men were higher at endline, but comparatively the increase was less than that among the men in the intervention arm communities, and the degree to which it was less was statistically significant for economic and emotional IPV. In interpreting this it is important to note that this study used repeat community surveys so it was not the same men and women responding at different time points.

It is not clear why we have different results for men and women. They were interviewed in different communities and were not in couples, so exactly the same results would not be expected, but we must consider how the intervention impacted one gender without as much effect on the other. It is possible that the women gave socially desirable responses and exaggerated change, however, if that is the case it is surprising that the change was seen in some IPV measures (sexual violence and controlling behaviour) and no significant reductions in all of them. The veracity of women’s reports are also supported by the correlated health outcome, depression, also reducing. An alternative explanation is that men gave socially desirable responses, exaggerating their use of violence in their efforts to be seen as tough men, or else that the intervention sensitised them more to their use of violence and the baseline under-reported it. These are both possible. Men’s levels of physical IPV reporting at baseline were lower than women’s, although their sexual IPV reporting levels were the same. A notable change in their reporting was in much higher levels of sexual IPV reported at endline, this was not at all similar to women’s reports. Given the discrepancy, our inclination is to trust women’s reports of violence experiences more than men’s as it is hard to know why women would exaggerate physical IPV at baseline, and it is more likely that women’s reports of sexual IPV are more reliable as they would be the ultimate arbitrators of whether they regarded an act as forced or otherwise.

Our findings showed relatively lower engagement and exposure to all intervention activities among men, with the lowest levels of reporting for one-on-one chats and home visits which allow better dialogue on key issues compared to other sensitisation activities. Given that exposure to the intervention is necessary to effect change, further research is needed to better understand the pathways to impact, with a goal of the intervention adaptation to enhance the effect on men. This is especially important, considering that lower participation by men in similar programmes is well documented (insert refs).

The intervention had an overall direction of positive impact on gender attitudes and norms except for social norms reported by men in communities that were perceived as more conservative. It is likely that the latter was due to changes in perceptions of communities after awareness raising i.e. previously men didn’t really reflect on how conservative their communities were. It does appear that improvements in gender attitudes and norms were not needed to effect changes in IPV and controlling behaviour experienced by women. It is possible that the direct counselling of couples and messages given by traditional leaders about violence were particularly impactful in the absence of evidence of overall gender attitudes and social norm change. Social norms take much time to change and it is possible that more impact could have been seen if there had been a longer intervention [21]. It is also possible that the measures of gender attitudes and social norms did not capture the full range of indicators of women’s empowerment in the community. The improvement in women’s depression may have resulted from actual support received from the COMBATs or the perceived societal support stemming from the visibility of VAW as a social issue created by the intervention. Women’s exposure to information on how to handle cases of VAW and the perceived affirmation of a woman’s power to seek help or redress could also have contributed to a reduction in depression.

The difference in men and women’s reports on male controlling behaviour may be reflective of differences in male and female perception of controlling behaviour among many other reasons including feelings of powerlessness [22]. The reduction in women’s report of male controlling behaviour suggests increased power and assertiveness among women in their relationships post-intervention. Conversely, the increased controlling behaviour reported by men may also be in response to the likely shift in power relations at home following intervention effects on women [22]. It is also possible that the increased controlling behaviour reported by men may be due to increased sensitisation regarding what constitutes controlling behaviour post-intervention.

This trial had some strengths and weaknesses worth mentioning. It involved very large samples of men and women in both pre-and – post-intervention surveys allowing for more precise estimates of outcomes and intervention effects. Secondly, the study outcomes were measured using standardised tools and thus comparable to work done in other settings. The ability of this trial to adjust for pre-intervention estimates in our assessment of intervention effects on study outcomes makes our findings more robust. The analysis of intervention effects was based on intention to treat which is a reflection of real-life situations and can be generalised to populations with similar settings. A possible weakness of this study is its reliance on self-reported study outcomes that can be accompanied by recall bias and social desirability, this is inevitable in violence research. We did not interview the same women and men on the two occasions so we cannot comment on the change in individuals’ behaviour but we have the benefit of reports not being due to multiple assessments.

There was one important area of difference between time points and arms for women, and this was in their levels of food insecurity. There was much less severe food insecurity reported in the post-intervention interviews in both study arms than in the pre-intervention interviews. At both time points, severe food insecurity was greater in the control arm than the intervention arm. Food security was identified at baseline as a risk factor for the experience of past year IPV, after adjusting for other variables, but it was not directly correlated, in that the intervention arm at baseline had less severe food insecurity than the control arm and higher reported physical IPV. An adjustment was made for food insecurity in the initial analysis but was excluded in the final model due to its nonsignificant effects on the estimates, and for the purpose of achieving an efficient model.

## Conclusion

Our evaluation has shown that the Rural Response System (RRS) intervention model has apparently had an impact on experience and perpetration of violence in rural communities in Central Ghana. This intervention worked through teams of trained and supported community activists, which seems to have critically provided support to couples experiencing violence as well as its work to sensitise communities to VAW. To our knowledge it is the third such intervention to work in this way, joining the ranks of SHARE and SASA! (discussed above) that showed evidence of impact in Uganda. The findings support the needs for further investment in delivering this intervention to rural communities in Ghana and suggest that much value could be gained from further research aiming to better understand the observed disparities in intervention effects on men and women and the pathways to change, as well as its impact in the context of scale-up.

## Paper context

Intimate partner violence is a global menace. We evaluated the Rural Response System intervention which uses community-based action teams to prevent violence against women and intimate partner violence in the Central Region, Ghana. Our findings indicate that the intervention reduced women’s experiences of intimate partner violence, depression, and partner controlling behavior, and some evidence of men’s reported reductions in the perpetration of intimate partner violence. The intervention warrants careful scale-up in Ghana and further research.

## Appendix 1. Outcome measures in the RSS trial

**Table:**

Outcome	Source	Indicator	Number of items	Method of scaling	Cluster-level summary	Hypothesised direction of change because of the intervention
**Primary outcomes**						
Physical IPV	WHO VAW scale (adapted) [23]	One or more episode of physical IPV in the past 12 months (men perpetrate; women experience)	5	Binary	Proportion	Decrease
Sexual IPV		One or more episode of sexual IPV in the past 12 months (men perpetrate; women experience)	3	Binary	Proportion	Decrease
Severe IPV		More than one episode of physical or sexual intimate partner violence in the past 12 months (men perpetrate; women experience)	8	Binary	Proportion	Decrease
**Secondary outcomes**						
Depressive symptomology (CESD)	Centre for Epidemiologic Studies Depression (CESD) scale [24]	Depression in the past week, continuous	20	Mean	Mean	Decrease
Controlling Behaviours	Modified Sexual Relationship Power (SRP) scale [25]	Experiences of control in primary relationships by a male partner (men perpetrate; women experience)	8	Mean	Mean	Decrease
Emotional/economic IPV	WHO VAW scale (adapted) [23]	One or more episodes of emotional IPV in the past 12 months (men perpetrate; women experience)	5	Binary	Proportion	Decrease
Gender attitudes	Modified Gender Equitable Men’s Scale (GEMS) [26]	Agreement with statements on gender attitudes (4 point Likert scale, ≥ more equitable)	8	Mean	Mean	Increase
Individual gender attitudes	Scale developed for Stepping Stones Creating Futures study [27]	Agreement with statements on gender attitudes (4 point Likert scale, ≥ more equitable)	9	Mean	Mean	Increase
Social norms	Scale developed for Stepping Stones Creating Futures study [27]	Agreement with statements on gender attitudes (4 point Likert scale, ≥ more equitable)	9	Mean	Mean	Increase
